# Identification of Disparities in Personalized Cancer Care—A Joint Approach of the German WERA Consortium

**DOI:** 10.3390/cancers14205040

**Published:** 2022-10-14

**Authors:** Florian Lüke, Florian Haller, Kirsten Utpatel, Markus Krebs, Norbert Meidenbauer, Alexander Scheiter, Silvia Spoerl, Daniel Heudobler, Daniela Sparrer, Ulrich Kaiser, Felix Keil, Christoph Schubart, Lars Tögel, Sabine Einhell, Wolfgang Dietmaier, Ralf Huss, Sebastian Dintner, Sebastian Sommer, Frank Jordan, Maria-Elisabeth Goebeler, Michaela Metz, Diana Haake, Mithun Scheytt, Elena Gerhard-Hartmann, Katja Maurus, Stephanie Brändlein, Andreas Rosenwald, Arndt Hartmann, Bruno Märkl, Hermann Einsele, Andreas Mackensen, Wolfgang Herr, Volker Kunzmann, Ralf Bargou, Matthias W. Beckmann, Tobias Pukrop, Martin Trepel, Matthias Evert, Rainer Claus, Alexander Kerscher

**Affiliations:** 1 Department of Internal Medicine III, Hematology and Oncology, University Hospital Regensburg, 93053 Regensburg, Germany; 2 Comprehensive Cancer Center Ostbayern, 93053 Regensburg, Germany; 3 Division of Personalized Tumor Therapy, Fraunhofer Institute for Toxicology and Experimental Medicine, 93053 Regensburg, Germany; 4 Institute of Pathology, Friedrich-Alexander University Erlangen-Nuremberg, University Hospital Erlangen, 91054 Erlangen, Germany; 5 Comprehensive Cancer Center Erlangen-EMN, 91054 Erlangen, Germany; 6 Institute of Pathology, University of Regensburg, 93053 Regensburg, Germany; 7 Comprehensive Cancer Center Mainfranken, University Hospital Würzburg, 97080 Würzburg, Germany; 8 Department of Urology and Pediatric Urology, University Hospital Würzburg, 97080 Würzburg, Germany; 9 Department of Medicine V, Hematology and Oncology, University Hospital Erlangen, 91054 Erlangen, Germany; 10 Bavarian Center for Cancer Research (BZKF), 91054 Erlangen, Germany; 11 Medical Faculty, Institute of Pathology and Molecular Diagnostics, University of Augsburg, 86156 Augsburg, Germany; 12 Comprehensive Cancer Center Augsburg, 86156 Augsburg, Germany; 13 Department of Hematology and Clinical Oncology, Medical Faculty, University of Augsburg, 86156 Augsburg, Germany; 14 Department of Internal Medicine II, University Hospital Würzburg, 97080 Würzburg, Germany; 15 Institute of Pathology, University of Würzburg, 97080 Würzburg, Germany; 16 Department of Gynecology and Obstetrics, University Hospital Erlangen, 91054 Erlangen, Germany

**Keywords:** precision oncology, MTB, patient access, cancer care, outreach, real world data, outcomes research

## Abstract

**Simple Summary:**

In Molecular Tumor Boards (MTBs), clinicians and researchers discuss the biology of tumor samples from individual patients to find suitable therapies. MTBs have therefore become key elements of precision oncology programs. Patients living in urban areas with specialized medical centers can easily access MTBs. Dedicated efforts are necessary to also grant equal access for patients from rural areas. To address this challenge, the four German cancer centers in Würzburg, Erlangen, Regensburg and Augsburg collectively measured the regional efficacy of their MTBs. By jointly analyzing the residences of all MTB patients, we uncovered regional differences in our mostly rural catchment area. Mapping and further understanding these local differences—especially the underrepresented white spots—will help resolving inequalities in patient access to precision oncology. Our study represents a hands-on approach to assessing the regional efficacy of a precision oncology program. Moreover, this approach is transferable to other regions and clinical applications.

**Abstract:**

(1) Background: molecular tumor boards (MTBs) are crucial instruments for discussing and allocating targeted therapies to suitable cancer patients based on genetic findings. Currently, limited evidence is available regarding the regional impact and the outreach component of MTBs; (2) Methods: we analyzed MTB patient data from four neighboring Bavarian tertiary care oncology centers in Würzburg, Erlangen, Regensburg, and Augsburg, together constituting the WERA Alliance. Absolute patient numbers and regional distribution across the WERA-wide catchment area were weighted with local population densities; (3) Results: the highest MTB patient numbers were found close to the four cancer centers. However, peaks in absolute patient numbers were also detected in more distant and rural areas. Moreover, weighting absolute numbers with local population density allowed for identifying so-called white spots—regions within our catchment that were relatively underrepresented in WERA MTBs; (4) Conclusions: investigating patient data from four neighboring cancer centers, we comprehensively assessed the regional impact of our MTBs. The results confirmed the success of existing collaborative structures with our regional partners. Additionally, our results help identifying potential white spots in providing precision oncology and help establishing a joint WERA-wide outreach strategy.

## 1. Introduction

Precision oncology has made immense progress in delivering novel therapies guided by molecular biomarkers to patients suffering from cancer. This was made possible by the rapid advancements in molecular diagnostics. While generating mutational profiles has become feasible and readily available, interpretation of mutational profiles and integration of molecular and clinical data for therapeutic recommendations is still a challenge. Molecular tumor boards (MTBs), usually located at tertiary care oncology centers, have therefore become ground-breaking and indispensable institutions for attributing specific drugs to suitable patients based on individual tumor biology [1,2,3,4]. Within MTBs, potential therapeutic strategies are discussed by clinicians, pathologists, and researchers such as molecular pathologists, human geneticists and bioinformaticians.

Despite their promises, there are concerns that precision oncology programs could exacerbate health disparities within societies by excluding patients in underserved regions—such as rural areas—and patients from underserved communities from these auspicious treatment options [5,6,7,8,9,10,11]. To address this potential threat, authorities from countries such as Japan and Norway have set up central infrastructure for implementing MTBs as core components of a nation-wide precision oncology ecosystem [12,13,14,15,16]. When setting up the “National Decade against Cancer” in 2020, the German Federal Ministry of Education and Research also decided to put special emphasis on providing equal access to precision oncology for all patients in Germany [17,18].

**Figure 1:**
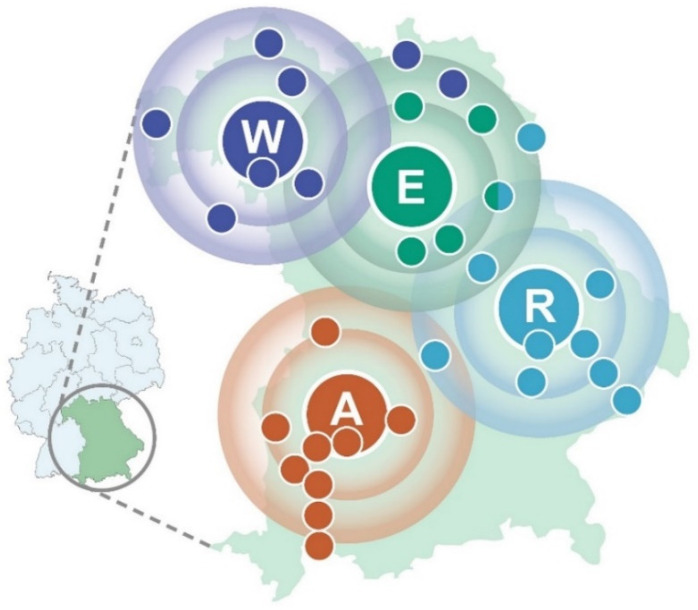
Regional catchment area of the WERA cancer center alliance—containing Würzburg (W), Erlangen (E), Regensburg (R), and Augsburg (A) as regional hubs with cooperating regional hospitals (colored smaller dots)—plotted on a map of the Federal State of Bavaria in Germany.

In line with this overarching objective, the four Bavarian tertiary care oncology centers in Würzburg (W), Erlangen (E), Regensburg (R), and Augsburg (A) founded the WERA Alliance to provide equal access to precision oncology for all patients from its mainly non-metropolitan/rural catchment area. The WERA Alliance, with its regional partner hospitals, covers a large part of the Federal State of Bavaria (Figure 1). These cooperation centers regularly refer patients to the university hospitals for trial inclusion, second opinions, tumor board discussions or specialized diagnostics. Regional network partners receive expertise (i.e., updated chemotherapy protocols, therapy recommendations, and joint clinical trial performance) in return. In addition to the four university medical centers, to date 108 regional partners of different sizes constitute the entire network. They encompass 49 office-based oncologists, 15 certified oncology centers, 44 regional hospitals, and 15 rehabilitation clinics. Additionally, the WERA Alliance currently cooperates with 95 patient advocacy groups.

Based on this expertise as a large clinical network of cancer care providers for a mainly rural catchment area, we assessed the status quo of our precision oncology program—specifically the outreach activity of our MTB program—which was established together with our regional partners. Therefore, all four WERA cancer centers jointly measured the regional impact of our MTBs by mapping the physical addresses of patients discussed in the years 2020 and 2021. In a further step, absolute patient numbers were weighted with local population densities in order to identify regions of our joint catchment area that were relatively underrepresented in WERA MTBs. Of note, members of the WERA Alliance are the only local providers of MTBs within the regional catchment area—thereby allowing the delineation of potential white spots in precision oncology.

## 2. Materials and Methods

### 2.1. Local Data Collection and Generation of a Merged Dataset

For this retrospective analysis, each site independently collected postal codes from physical addresses (at time of board discussion) of all MTB patients at the university hospitals of Würzburg, Erlangen, Regensburg, and Augsburg in the years 2020 and 2021.

According to our harmonized MTB standard operating procedure, each WERA site included patients suffering from an advanced tumor disease with limited or no treatment options according to guidelines. Of note, individual MTB presentation was independent from financial reimbursement issues.

Data were provided by local tumor registries of each university hospital and, if necessary, local hospital information systems. After generating regional coverage data for each WERA center, anonymized postal code information (i.e., the number of MTB patients living in a certain postal code area) were merged in a centralized database located at Würzburg’s tumor registry (“Krebsregister CCC Mainfranken”). The study was conducted according to the guidelines of the Declaration of Helsinki and approved by the Ethics Committee of the University of Regensburg (Molecular Tumor Board Registry Study, protocol code 20-1682-101). Due to the retrospective nature and the exclusive usage of anonymized data, this multi-center study was also in accordance with local GDPR and the Bavarian Hospital Act (“Bayerisches Krankenhausgesetz”). Two researchers (F.L. and A.K.) independently supervised the process of data merging and subsequently performed analyses of the joint dataset.

### 2.2. Analysis of the Merged Dataset and Illustration of Results

German population data for respective postal code areas were downloaded from an open-source database (https://www.suche-postleitzahl.org/downloads (accessed on 5 May 2022)—combining German postal code information provided by OpenStreetMap (https://www.openstreetmap.org (accessed on 5 May 2022) with population data from German statistical offices within the “Zensus 2011” initiative (https://www.zensus2011.de (accessed on 5 May 2022). For weighting patient numbers with local population densities, we determined the number of MTB patients per 100,000 inhabitants (in the following termed: local patient representation). Moreover, median values for each WERA site were calculated to assess the regional dispersion of absolute and relative patient numbers per postal code area. Absolute as well as relative MTB patient numbers per postal code area were plotted on a map of Southern Germany (Figure S1). Data merging, curation and calculations were performed with Microsoft^®^ Access^®^ 2016 (version 16.0.5224.1000, Redmond, WA, USA), visualization and illustration (including mapping of absolute and relative patient numbers per postal code area) were performed with QGIS, an open-source graphical information system (QGIS Development Team; under license of GNU General Public License, Version 3.26.3, Gary E. Sherman et al., Boston, MA, USA).

## 3. Results

Our regional analysis is based on official postal code areas, with 8170 areas covering Germany. WERA MTB patients included in this study came from 649 different postal code areas, representing 7.94% of all German districts.

### 3.1. Characterizing MTB Patients from Würzburg, Erlangen, Regensburg and Augsburg

**Figure 2:**
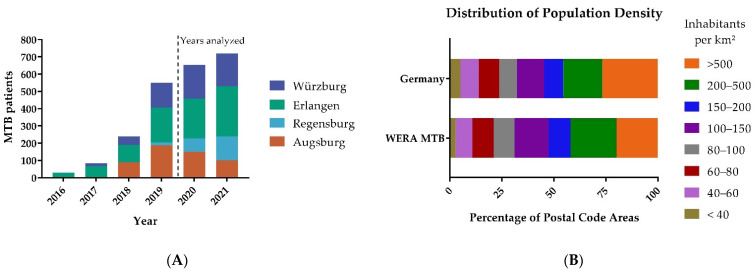
(**A**) Patients included in WERA MTBs between 2016 and 2021. Erlangen and Würzburg began recruiting patients in 2016 and 2017, respectively. Augsburg and Regensburg followed in 2018 and 2019, respectively. In 2020 and 2021, all four centers recruited substantial patient numbers. Therefore, these two years were analyzed. (**B**) Distribution of population density among WERA MTB patients compared to the general population density of Germany.

MTBs in Erlangen and Würzburg began recruiting patients earlier than MTBs in Augsburg and Regensburg. Within one year, all four sites were recruiting substantial numbers of patients in their catchment area as shown in Figure 2A. In order to mitigate a potential bias resulting from Augsburg and Regensburg still establishing their MTB workflows, we chose to pool the data of 2020 and 2021. Detailed patient numbers for each WERA MTB are summarized in Table 1.

In total, our study analyzed the regional origin of 1374 MTB patients, with 385 patients from Würzburg and 521 from Erlangen. Regensburg and Augsburg contributed 217 and 251 MTB patients, respectively. While there was a reduction in MTB cases in Augsburg, which can be attributed to the COVID-19 pandemic, we could increase the overall number of patients in precision cancer care.

In a further step, we calculated (absolute) patient numbers for each postal code area of the WERA outreach. Maximum local patient numbers per postal code area ranged from 7 (Regensburg) to 19 (Erlangen). After dividing patient numbers by local population per postal code area, maximum local patient representation ranged from 111.23 pts./100,000 inhabitants (Regensburg) to 294.99 pts./100,000 inhabitants (Erlangen). Regarding local patient numbers, all four sites displayed a median value of 1—thereby confirming the high resolution of our postal code-driven approach. For patient representation per postal code area, median values ranged between 18.93 pts./100,000 inhabitants (Regensburg) and 27.64 pts./100,000 inhabitants (Augsburg). Altogether, comparable median values of patient representation per postal code area across all four sites mirror successful clinical networking with regional partners. Low median values might also underline significant outreach activities; patients from many postal code areas are referred to our comprehensive cancer centers, rather than many patients from few postal code areas. We also compared the distribution of the population density in areas with MTB patient referral to the distribution of the population density in Germany as shown in Figure 2B. In comparison to Germany, we found a similar distribution of population density. We also observed marked differences in particularly sparsely populated areas and the most densely populated areas.

**Figure 3:**
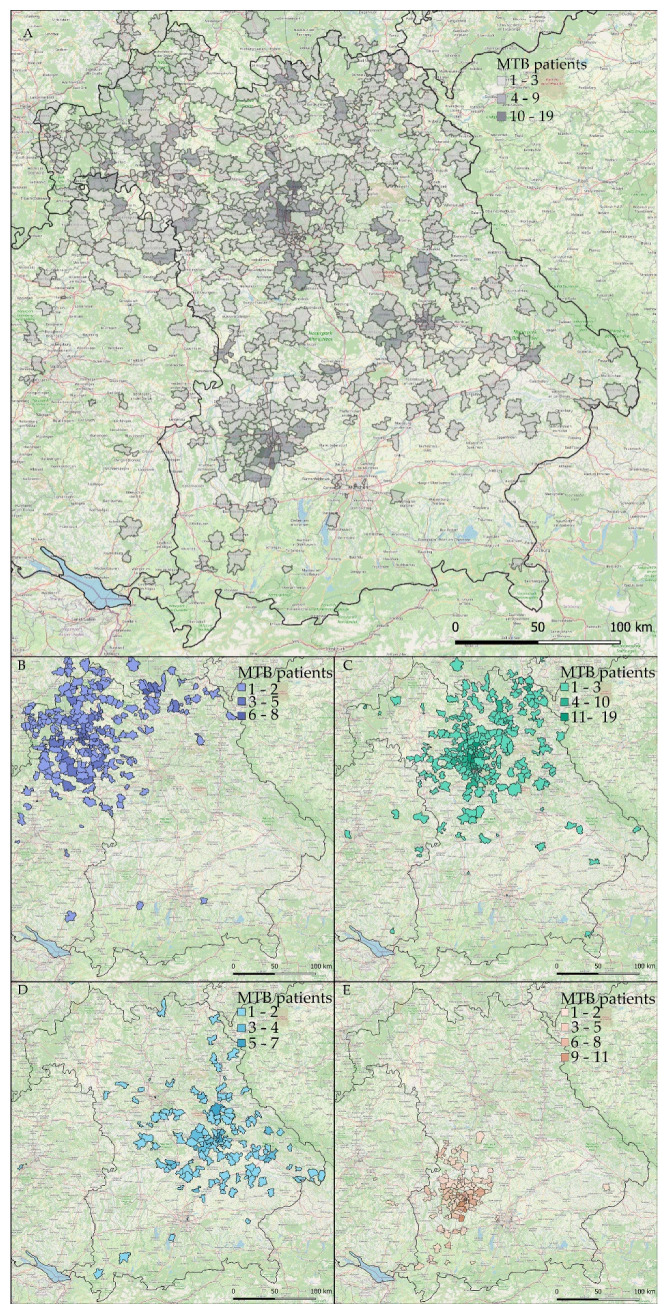
Absolute numbers of patients discussed in WERA MTBs in the years 2020 and 2021. Results are plotted on a map of the Federal State of Bavaria and surrounding regions: (**A**) combined patient number of all four centers; (**B**–**E**) individual patient number of each center; (**B**) Würzburg; (**C**) Erlangen; (**D**) Regensburg and (**E**) Augsburg.

To further characterize and illustrate our current regional impact, we plotted absolute numbers of MTB patients from all four sites on a map of Southern Germany as illustrated by Figure 3.

At first sight (Figure 3A), WERA MTBs already cover a substantial part of the joint catchment area shown in Figure 1. Moreover, the WERA sites complement each other well in terms of regional distribution (Figure 3B–E). Of note, MTB patients did not exclusively live in the Free State of Bavaria, but also in the neighboring Federal States of Baden-Württemberg (e.g., regions close to Heilbronn and Bad Mergentheim), Hesse (regions around Frankfurt/Main and Fulda), and Thuringia (Sonneberg region). Peaks in absolute patient numbers were seen for regions close to the four WERA university hospitals, which reflects the substantial part of in-house MTB patients previously receiving (routine) cancer care at one of the WERA university medical centers.

However, we also identified clusters of patients beyond urban areas, for example, Kulmbach in the north-eastern part of Bavaria and the region around Straubing, Deggendorf, and Passau (partners of CCC Ostbayern) in the eastern part of Bavaria.

### 3.2. Relative Regional Representation of Cancer Patients in WERA MTBs

**Figure 4:**
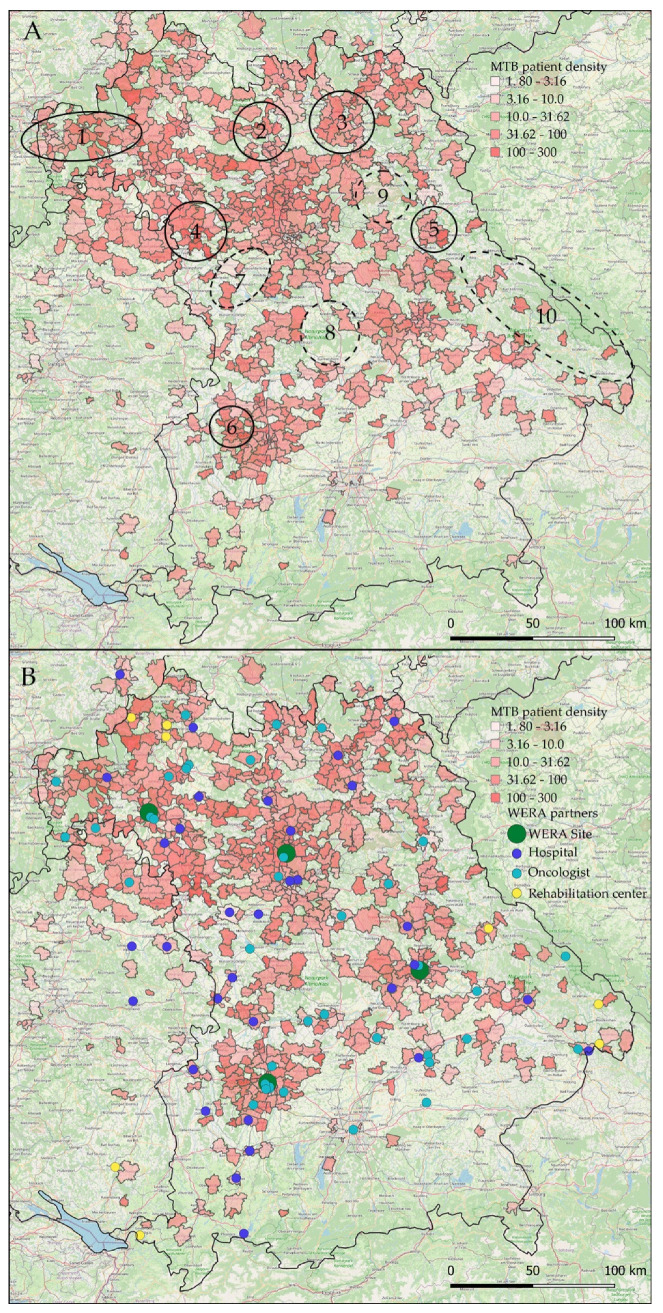
Local representation of patients discussed in WERA MTBs in the years 2020 and 2021. Absolute numbers were divided by local population densities (MTB patients per 100,000 inhabitants): (**A**) MTB patient density of all four combined centers, closed circles indicate “hot spots” with high patient density, dashed circles indicate “white spots” with low patient density; (**B**) patient density and WERA collaboration network partners, illustrating outreach efforts of the consortium. Some collaboration partners had identical postal codes and are shown only once.

To account for overrepresentation of urban areas with higher population densities and to allow for a more differentiated view on our regional impact, we divided absolute MTB patient numbers by local population for each postal code area. This data transformation step highlighted existing networking structures of each WERA site by revealing “novel” peaks in rural areas and in the periphery of our catchment area (Figure 4 and Figure S2). Successful outreach activity was also reflected by similar measures of dispersion across all WERA sites regarding median local patient representation (Table 1).

Specifically, this step allowed us to precisely locate postal code areas strongly represented in WERA MTBs during the recent two years, as well as areas which were underrepresented at the same time. As shown in Figure 4A (closed circles), postal code areas close to Aschaffenburg (No. 1) in the north-western part of Bavaria displayed high counts in local patient representation. Moreover, WERA MTBs also discussed a high number of patients living in areas such as Bamberg (No. 2), Kulmbach (No. 3), and Rothenburg ob der Tauber/Bad Windsheim (No. 4). Regarding strongly represented areas close to the WERA cancer centers in Regensburg and Augsburg, we identified the rural area around Neunburg vorm Wald (No. 5) and the Günzburg/Burgau region (No. 6), respectively.

As also illustrated in Figure 4A (dashed circles), we could additionally delineate postal code areas which were underrepresented in MTBs. We also considered the surrounding area. When there was a marked decline in MTB patients compared to neighboring regions, we still considered this area underrepresented. This was the case for the region close to Ansbach (No. 7) and the rural area between Nuremberg and Ingolstadt (No. 8). Interestingly, area No. 9 contains the military training ground Grafenwöhr, basically representing an uninhabited region. The Grafenau region (No. 10) as well as neighboring regions close to the Czech border also emerged as white spot areas, possibly because only few practices specialized in hematology or oncology are situated in this rural area within a certain radius.

Having a closer look at the location of regional healthcare providers as potential cause of local MTB representation, we added our regional partner network to our graphical analysis (Figure 4B). As outlined above, regional “hot spots” were frequently located in vicinity to established partner sites—such as the Spessart region close to Aschaffenburg (closed circle No. 1 in Figure 4A) or Kulmbach (No. 3). Moreover, low patient representation often went along with a weak regional coverage in terms of network partners, especially regarding the underserved regions No. 8 and 9 in Figure 4A.

## 4. Discussion

Substantial parts of health disparities research in oncology examine distance from healthcare providers as a crucial obstacle in cancer care; importantly, the coverage of rural areas poses a challenge not only for low- and middle-, but also high-income countries [19,20,21]. Given that MTBs together with related oncologists, pathologists, human geneticists and researchers are frequently located at tertiary care cancer centers in urban regions, overcoming geographical distance will at least maintain its relevance. Therefore, clinical networks between local healthcare providers and cancer centers as well as novel technical solutions (e.g., telehealth) are needed. In this study, we aimed to assess the current “regional impact” of our clinical MTB network by merging patient care data from our four WERA MTB sites.

### 4.1. Gaining Insight through Cooperation and Joint Data Analysis

Various studies previously analyzed the organizational and technical setup of MTBs as well as its impact on clinical decision-making and its benefit for cancer patients [1,22,23,24,25,26,27]. Additionally, researchers examined findings and recommendations of MTBs, which discussed patients from community-based oncology practices [2,28,29,30]. However, there is limited evidence in terms of the regional impact of MTBs for a distinct catchment area. In our retrospective analysis, we therefore investigated the regional distribution of patients discussed in the MTBs of the Bavarian university hospitals of Würzburg, Erlangen, Regensburg, and Augsburg, together constituting the WERA Alliance and covering a large and coherent catchment area of around eight million inhabitants.

By jointly investigating our patient-centered care, we assessed our current impact on precision oncology within the WERA-wide catchment area. Regarding absolute patient numbers, the highest peaks were seen for areas close to our four university hospitals. This result was not surprising, as it reflects high numbers of cancer patients primarily treated at our tertiary care centers as well as higher population densities in these metropolitan areas. However, we also found substantial peaks of MTB patient numbers in rural areas, which reflect existing and successful collaboration with regional health care providers. Altogether, mapping MTB patients from all four tertiary care cancer centers illustrated the existing clinical network with regional healthcare providers and confirmed the widespread regional impact of the WERA Alliance.

### 4.2. Identifying Potential White Spots in Precision Oncology

To account for heterogeneous population densities across the WERA catchment area, relative representation of a certain postal code area within MTBs was defined as patients per 100,000 inhabitants. This approach specifically highlighted established networking with regional partner hospitals and oncologists in private practices. These results underline that regional networks substantially increase treatment options for patients with cancer living in rural areas. We strongly believe that precision oncology programs need to be embedded into widespread clinical networks, as they require awareness that can only be successfully sustained by broader cooperation.

In contrast to rural areas strongly represented in WERA MTBs, we also identified potential white spots within our catchment area, i.e., regions which were underrepresented in MTBs in the years 2020 and 2021. A thorough look at each of these white spots revealed some potential reasons for the underlying causes of this statistical underrepresentation. In general, there could be a lack of information and awareness regarding the benefit of precision oncology programs among both health care providers and patients. Moreover, underrepresentation in MTBs could be caused or at least worsened by the declining number of oncologists/hematologists working in private practices in rural areas, a trend, which has increased in Germany over the last years. Another reason for potential white spots—demonstrated by the military training ground in Grafenwöhr—could be sparse overall population of certain regions. Demographic and geographic features of a given region can have significant impact on such an analysis and need to be taken into account. Moreover, white spots in our WERA-wide analysis might be covered by MTBs of different cancer centers. In our case, physicians might have sent their patients to one of the two MTBs at the university hospitals of Munich, which currently are not part of this analysis. As a consequence, each white spot candidate in our catchment area requires an in-depth analysis—above all to identify regions where a lack of information and awareness among healthcare providers as well as patients causes underrepresentation.

In our view, being able to locate these potentially underserved regions clearly shows the benefit of our joint approach, as no single-center analysis can address such a research question. These “negative results” will support WERA ’s precision oncology policy by directing our outreach measures specifically towards underserved areas.

### 4.3. Limitations and Future Directions

Our study has several limitations. Firstly, we used a simplistic model with the basic assumption of equally distributed cancer incidences across our catchment area. More specifically, we did not account for differences in cancer risk factors such as the age of the local population. However, given that MTBs cover all tumor entities, and the influence of certain risk factors is not equally distributed between cancer entities, we decided against stratifying for these risk factors. Secondly, this analysis did not define cut-off values for marking regions as white spots. Any attempt to quantify a white spot would require standardization of each CCC ’s patient numbers, catchment area, and patient referrals. Currently, our joint dataset does not have the depth to enable such a detailed analysis. Additionally, with this approach, we aimed to gain an overview of our catchment area and to concert our future outreach activities. In future, we will further elaborate our analyses as our data sets gain more detailed information. Additionally, as already stated above, we cannot rule out that patients from underrepresented regions are sent to another tertiary care oncology center outside the WERA Alliance, especially in the periphery of our catchment area. Yet, such a systematic bias appears improbable for the inner part of our catchment area constituted by our four neighboring centers. Lastly, we should state that patients discussed within MTBs clearly represent the “tip of the iceberg” in precision oncology, as many targeted therapies are also discussed and attributed within organ-specific tumor boards. Pioneering studies in lung cancer patients uncovered the value of targeted agents, demonstrating the value of structured screening for actionable mutations in cancer patients. Many of these alterations (e.g., EGFR. ALK, ROS) have already found their way into standard of care procedures [31]. Other entities have followed—for example, therapeutic implications of BRAF (B-Raf Oncogene) mutations in patients suffering from malignant melanoma [32] are usually discussed within the dermatologic tumor board. Similarly, alterations of the DNA repair pathway in prostate cancer tissue are usually discussed within the urologic tumor board [33].

Due to these limitations, our multi-center study clearly has an exploratory and descriptive character. We generally have to concede that we are just beginning to understand the influence of our local healthcare provider network; while we have detected several highly active partner regions, we also detected underserved regions close to network partners. These results imply that other determinants such as social networks must be considered in future. Interestingly, some regions not directly covered by network partners also emerged as highly represented, which again could mean that other crucial determinants are currently not considered. This further underlines that analyses such as this can help to pinpoint problems in our catchment area. Causes for white spots are multifaceted and need to be addressed individually. At the same time, knowing where your problems are frees resources elsewhere that can be redirected to improve MTB coverage where needed most. Yet, we are convinced that it is a further step to harmonize our outreach policy and to get a deeper understanding of what is needed to provide comprehensive precision oncology programs for our rural catchment area. In future, we will refine our analysis by considering local cancer incidences, MTB-specific distributions of cancer entities, and local demographic factors. Measures to improve our joint precision oncology program will include the integration of patient representatives and advocacy groups to raise awareness in the patient community. Moreover, information campaigns together with local healthcare providers and medical associations could provide valuable feedback on how to further improve accessibility in rural areas. This will help to allocate resources efficiently towards areas with the biggest need, ultimately helping to limit health care costs and avoiding unnecessary and redundant infrastructure. Finally, establishing a standardized cohort of WERA MTB patients together with a harmonized clinical follow-up will allow us to gain deeper molecular insights and to demonstrate the clinical benefit for patients analyzed and discussed within MTBs, which in turn will raise awareness for referring suitable patients to our boards. We also believe that the straightforward approach presented in this work—merging care data from all relevant health care providers of a given catchment area in order to identify white spots—could easily be transferred to other German and European regions.

## 5. Conclusions

Merging patient data from four neighboring tertiary care cancer centers located in Southern Germany, we comprehensively assessed the regional impact of our MTBs. The results confirmed the success of existing collaborative structures with our regional partners. Additionally, our study identified potential white spots in terms of access to precision oncology, i.e., specific areas, which were underrepresented in our multi-center retrospective analysis of MTB patients. These negative results will further guide our regional outreach activities in order to provide equal access to precision oncology for all patients of our joint catchment area, especially those living in rural areas.

## Figures and Tables

**Table 1:** Patient numbers of our joint study cohort for each WERA MTB. In order to obtain local patient representation, absolute patient numbers (*n*) were divided by local population (*n*/100,000 inhabitants). IQR: Interquartile range.

WERA Site	Absolute Patient Numbers (*n*)	Max. Patient Numbers Per Postal Code Area (*n*)	Max. Patient Representation Per Postal Code Population (*n*/100,000)	Median Patient Numbers Per Postal Code Population (*n*); [IQR]	Median Patient Representation Per Postal Code Population (*n*/100,000); [IQR]
Würzburg	385	8	194.17	1; [1]	24.35; [32.11]
Erlangen	521	19	294.99	1; [1]	27.39; [32.76]
Regensburg	217	7	111.23	1; [1]	18.93; [24.21]
Augsburg	251	11	251.57	1; [2]	27.64; [32.83]

## Data Availability

The data for this study are available on request.

## Supplementary Materials

The following supporting information can be downloaded at: https://www.mdpi.com/article/10.3390/cancers14205040/s1. Figure S1: (A) Map of Bavaria and Southern Germany; (B) population density shown as 100,000 inhabitants per square kilometer and postal code area; Figure S2: distribution of WERA MTB patients in 2020 and 2021 per 100,000 inhabitants.
