# Supplementary material for: Identification of Disparities in Personalized Cancer Care—A Joint Approach of the German WERA Consortium
Source: Cancers (Basel). 2022 Oct 14;14(20):5040. doi: 10.3390/cancers14205040 (PMC9600149; doi:10.3390/cancers14205040)
Supplement: Supplementary file 1 [file cancers-14-05040-s001.zip › cancers-1853938-supplementary.pdf]

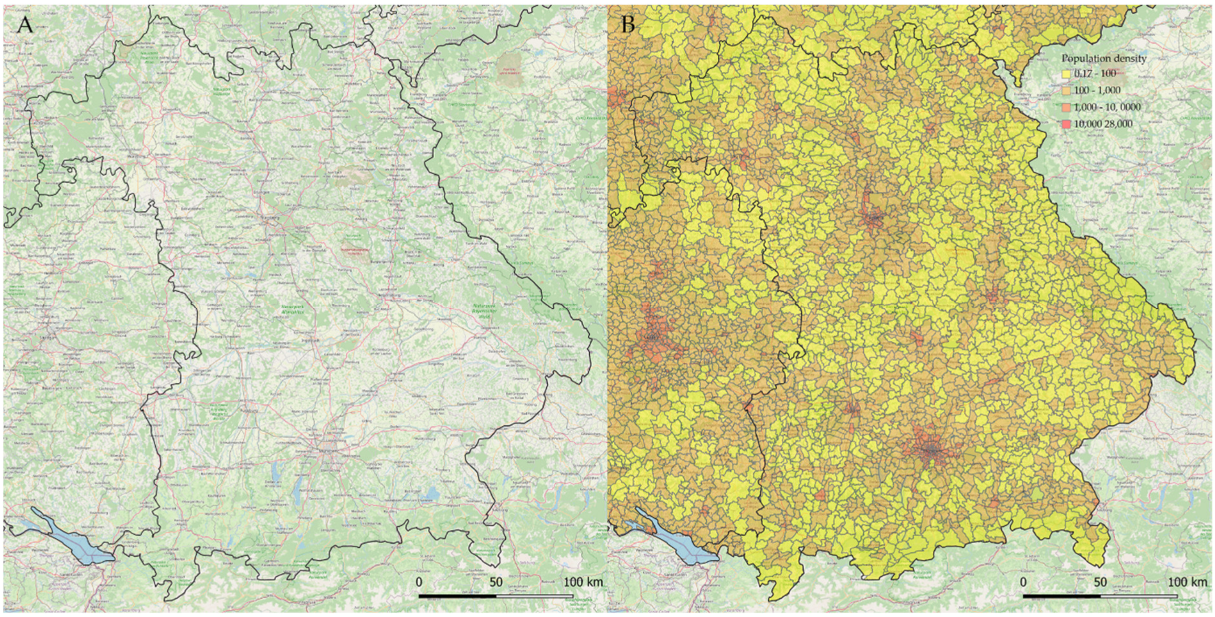

Figure S1. (**A**): Map of Bavaria and Southern Germany; (**B**): population density shown as 100,000 inhabitants per square kilometer and postal code area.

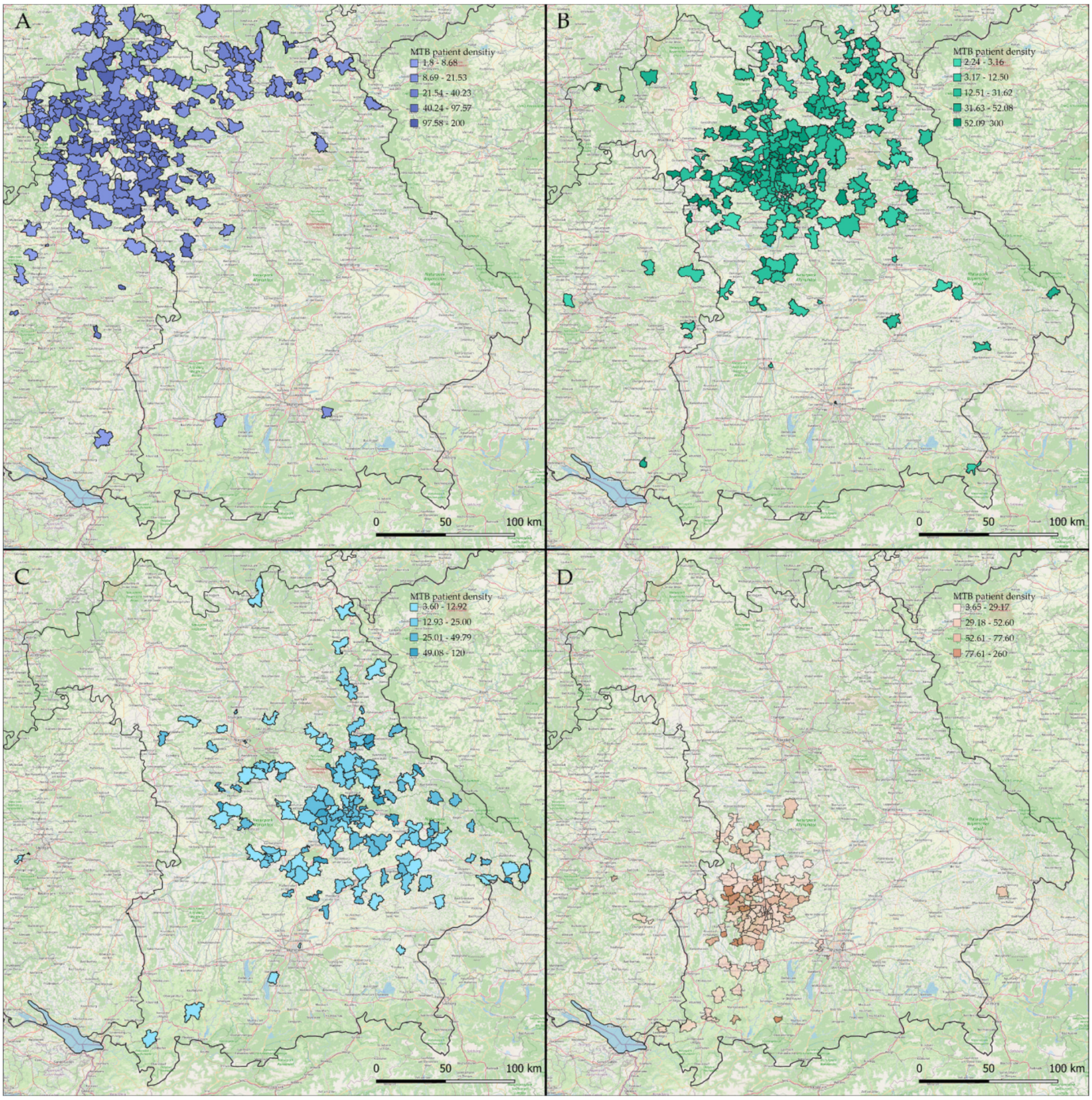

Figure S2. Distribution of WERA MTB patients in 2020 and 2021 per 100,000 inhabitants; (**A**–**D**) individual patient density for each center, (**A**) Würzburg, (**B**) Erlangen, (**C**) Regensburg, (**D**) Augsburg.
